# Positivity of anti-Zika virus (ZIKV) antibodies among patients undergoing ART in São Paulo State, Brazil (2016-2024): a retrospective record analysis

**DOI:** 10.5935/1518-0557.20260041

**Published:** 2026

**Authors:** Denise Maria Christofolini, Clara Helena Vicentini Ferreira do Valle, Guilherme Bom Oliveira, Laércio da Silva Paiva, Bianca Bianco, Caio Parente Barbosa

**Affiliations:** 1 Centro Universitário FMABC, Departamento de Morfologia e Fisiologia, Disciplina de Biologia Celular e do desenvolvimento, Santo André, São Paulo, Brazil; 2 Instituto Ideia Fértil de Saúde Reprodutiva, Santo André, São Paulo, Brazil; 3 Centro Universitário FMABC, Graduação, Santo André, São Paulo, Brazil; 4 Centro Universitário FMABC, Departamento de Saúde da Coletividade, disciplina de Saúde da Coletividade, Santo André, São Paulo, Brazil; 5 Centro Universitário FMABC, Departamento de Patologia, Disciplina de Genética, Santo André, São Paulo, Brazil; 6 Centro Universitário FMABC, Departamento de Saúde da Coletividade, disciplina de Saúde Sexual e Reprodutiva e Genética de Populações, Santo André, São Paulo, Brazil

**Keywords:** ZIKV, microcephaly, assisted reproductive technology, antibodies

## Abstract

**Objectives:**

To evaluate the presence of anti-ZIKV IgM and IgG antibodies in couples undergoing Assisted Reproduction Techniques (ART) and to examine reported ZIKV infections among women of reproductive age in São Paulo state.

**Methods:**

A retrospective cohort analysis was performed using medical records of ART patients consecutively tested for ZIKV between March 2016 and June 2024 at a private ART center. Testing involved a rapid immunochromatographic assay, with positive results confirmed by PCR. According to ANVISA regulations, anti-ZIKV IgG testing was required from 2016 to 2022, while both IgG and IgM testing became mandatory from 2022 to 2024. Additionally, ZIKV notification data from DATASUS for metropolitan cities near the ART center were collected and compared with positivity rates observed in the cohort.

**Results:**

Anti-ZIKV IgM positivity was 0.18% (1/569 female patients; 95% CI ≈ 0.00-0.98%), while anti-ZIKV IgG positivity was 2.1% (2/96 tested; 95% CI ≈ 0.25-7.32%), indicating limited prior exposure. DATASUS data showed a significant decline in ZIKV cases in São Paulo state, from 10,455 cases in 2016 to 228 in 2024. In cities surrounding the ART clinic, reported cases decreased from 73 to 1 during the same period. Among women of reproductive age, 42 cases were reported in 2016 and none in 2024.

**Discussion:**

Conducting widespread population testing creates logistical and financial challenges. In this ART group, ZIKV positivity was rare (<1%), with about 2% showing signs of previous exposure, and most patients came from regions with low rates of ZIKV-related microcephaly.

**Conclusion:**

Positivity for anti-ZIKV was low among patients on ART.

## INTRODUCTION

Zika virus (ZIKV) is a flavivirus from the Flaviviridae family, isolated in 1947 from the blood of a sentinel rhesus macaque placed in the Zika Forest of Uganda, with unknown effects on human health at the time (Dick *et al*., 1952). Human illness caused by ZIKV was first recognized in Nigeria in 1953, when viral infection was confirmed in three ill individuals (MacNamara, 1954).

The virus spread across various countries and continents, and in March 2015, the first cases in the Americas were reported in Salvador, Bahia, Brazil (Zanluca *et al*., 2015; Campos *et al*., 2015). Epidemiological data show that ZIKV reached 14 Brazilian states within seven months (Cardoso *et al*., 2015). In September of that year, Brazilian physicians and researchers first linked microcephaly in newborns to areas with ZIKV outbreaks (Schuler-Faccini *et al*., 2016). This link became especially clear when over 4,300 microcephaly cases were reported by February 2016. Today, ZIKV ranks among the top global causes of congenital malformations, leading the World Health Organization (WHO) to declare it a Public Health Emergency of International Concern in 2016 (Gulland, 2016).

ZIKV transmission typically occurs through a human-mosquito-human cycle (Gubler, 2002). Notably, ZIKV can also spread via non-vector routes. Studies have confirmed vertical transmission during pregnancy, sexual contact, and blood transfusion (Foy *et al*., 2011; Venturi *et al*., 2016; Besnard *et al*., 2014; Musso *et al*., 2015).

Considering sexual transmission, male to female (Foy *et al*., 2011; Venturi *et al*., 2016; D’Ortenzio *et al*., 2016) and male to male (Deckard *et al*., 2016) transmission have been reported, and transmission through vaginal, anal (Deckard *et al*., 2016), and oral sexual intercourse has been implicated (D’Ortenzio *et al*., 2016). Additionally, replicative viral particles and viral RNA have been identified in sperm, and viral RNA has been detected up to 62 days after the onset of symptoms (Mansuy *et al*., 2016).

Thus, the rise in microcephaly cases and concerns about sexual transmission led to increased discussions on ZIKV testing for prospective pregnancy patients. As a result, in March 2016, the Collegiate Board of ANVISA - Brazil’s Health Regulatory Agency - revised RDC No. 23/2011, making laboratory screening for the Zika Virus (including anti-Zika IgM and molecular tests) mandatory for sperm, oocyte, and tissue donors, in addition to existing tests such as syphilis, hepatitis, HIV, and HTLV. This aimed to ensure safety in assisted human reproduction (ART) procedures in Brazil, especially amid the Zika epidemic (RDC nº 72/2016) (ANVISA, 2016). For men with reactive or inconclusive results, molecular biology testing (PCR) should also be performed on semen samples. Additionally, couples undergoing ART are not allowed to continue treatment for 30 days if they test positive for ZIKV, even if they are asymptomatic (RDC nº 72/2016) (ANVISA, 2016).

In December 2022, the screening criteria for the Zika virus, including serological and molecular tests and periods of ineligibility for gamete donation, were maintained and updated. However, the requirement for ZIKV testing for personal use became optional (RDC n^o^ 771/2022) (ANVISA, 2022).

To date, to our knowledge, no assessment has been made of the effectiveness of this testing protocol. This study is the first of its kind in Brazil, providing essential data on ZIKV exposure among the reproductive-age population in São Paulo. For comparison, we also examined the number of reported ZIKV infection cases across various cities in the São Paulo metropolitan area to identify potential regional similarities and differences.

## OBJECTIVES

1. To evaluate the positivity of ZIKV infection in women undergoing assisted reproduction and their partners between January 2016 and June 2024.
2. To describe the number of ZIKV infection cases reported among women aged 15 to 39 years, using DATASUS data, across various cities in the state of São Paulo.

## MATERIALS AND METHODS

The initial phase of this study involved a retrospective cohort of patients who were consecutively evaluated and selected from individuals undergoing assisted reproductive technology (ART) at the Ideia Fértil Institute, a well-known private fertility clinic in Brazil. This clinic has three locations in the São Paulo metropolitan area (Santo André, Moema, and Guarulhos), with data gathered from detailed medical records.

### Inclusion and Exclusion Criteria

Eligibility required ZIKV testing from January 2016 to June 2024 for patients of all ages, sexes, and clinical profiles. Cases with incomplete test data were excluded, and for individuals with repeated testing, only the first valid result per episode was used to prevent duplication in positivity calculations.

### Testing Protocols

Anti-ZIKV antibody screening was part of the standard protocol before oocyte retrieval or embryo transfer, requiring rapid IgG/IgM tests for all patients involved in personal treatments or gamete donation. From March 2016 to December 2022, IgM testing alone was sufficient per RDC 72/2016; since then, RDC 771/2022 mandated simultaneous IgG assessment.

### Specific Procedures for Oocyte Donation

Fresh oocyte donors had to test negative for ZIKV within five days before donation. Protocols extended to partners, including same-sex couples, with negativity required for male gamete donation or inter-female embryo transfers.

### Analytical Methods

A cross-sectional design evaluated IgG/IgM seroprevalence using the Advagen Biotech Zika IgG/IgM Ab LF rapid test (SKU: ZKV-001-20; ANVISA registration no. 81472060012), an immunochromatographic assay with 97.4% sensitivity and 97.7% specificity for IgG, and 96.3% sensitivity and 97.3% specificity for IgM. Positive rapid tests were confirmed with PCR, and only verified positives were included.

### Data Collection

For female patients, variables included initial ZIKV serology (test dates, IgM/IgG outcomes, methodologies) and follow-up tests for subsequent procedures (oocyte donation or embryo transfer); male partner data included test timing and IgM results.

### Ethical Considerations

The protocol received approval from the Centro Universitário FMABC Ethics Committee (CAAE: 6.692.801). Anonymized analyses used registry numbers to protect confidentiality across all three sites, ensuring better geographic and demographic representation.

### Study Design Rationale

This multicenter framework enhanced validity by including diverse patient groups under consistent protocols, supporting reliable ZIKV positivity estimates in ART populations. Primary endpoints consisted of IgM and IgG point positivity at testing. Reporting followed STROBE (Strengthening the Reporting of Observational Studies in Epidemiology) guidelines.

### DATASUS Data Collection

Secondary data were systematically extracted from Zika virus case records accessible via DATASUS, utilizing the Notifiable Diseases Information System (SINAN) (DATASUS, 2024) through the TABNET platform of the Brazilian Ministry of Health. These publicly available data can be retrieved from **https://datasus.saude.gov.br/informacoes-de-saude-tabnet/**. Queries were configured with rows representing São Paulo state municipalities, columns indicating notification year, content as total notified cases, and the period covering 2016 to 2024; filters focused on sex and age group, particularly women of reproductive age (15-39 years). Extraction took place in August 2024, with data exported in CSV format for further processing and statistical analysis, following TABNET’s standardized interface.

### Data Stratification and Validation

Stratification enabled analyses by notification year, sex, age group, and geographic units, including São Paulo state and selected municipalities (São Paulo, Santo André, São Bernardo do Campo, São Caetano do Sul, and Diadema). Extraction was conducted independently by two researchers, with discrepancies adjudicated by a third to ensure consistency and enhance internal validity.

### Analytical Implications

This approach enabled the analysis of Zika virus incidence trends and spatial distribution throughout the study period, focusing on demographic variables in reproductive-age women due to their epidemiological relevance. Combining these public health data with clinical records from the Ideia Fértil Institute placed institutional prevalence in a broader municipal and state context, enhancing the strength of conclusions through integrated individual and population-level insights.

### Statistical Analysis

Positivity rates for IgG and IgM antibodies were computed independently using basic statistical techniques, with 95% confidence intervals determined through the Clopper-Pearson exact binomial method in Stata software (version 18.0). These positivity rates and other key findings from our study were then qualitatively compared with epidemiological data from the DATASUS database. This comparison helped us place our results within the larger context of ZIKV positivity among women of reproductive age across different regions of São Paulo state.

The analysis focused particularly on identifying potential correlations or discrepancies between our clinical data and the population-level statistics from DATASUS. By examining these patterns, we were able to assess regional differences in ZIKV exposure and evaluate how our findings from assisted reproduction patients matched or differed from general population trends. This indirect comparison provided valuable insights into the epidemiological profile of ZIKV infection across different areas of São Paulo, while also helping to validate our clinical observations against established public health data.

Public macrodata extracted from the DATASUS system, through the Notifiable Diseases Information System (SINAN, 2024), accessed via the TABNET platform, were used. The data cover the period from 2016 to 2024 and are stratified by sex and location, including the State of São Paulo, the capital (São Paulo), and the municipalities of São Bernardo do Campo (SBC), São Caetano do Sul (SCS), Santo André (SA), and Diadema (DD). The primary measure of analysis was the frequency of reported cases. Data was collected from July to August 2024.

The combination of our institutional results and the DATASUS data provided a more thorough understanding of ZIKV positivity trends in the state.

## RESULTS

Considering the first part, this study analyzed medical records from 569 couples undergoing ART between January 2016 and June 2024 to assess ZIKV positivity in this population. Data analysis revealed a 0.18% (95% CI ≈ 0.00-0.98%) IgM positivity rate (1 out of 569 female patients), and a 2.1% (95% CI ≈ 0.25-7.32%) IgG positivity rate (2 out of 96 tested). None of the 368 tested partners showed IgM positivity (0.0%, 95% CI ≈ 0.00-1.00%). The patient who tested positive in her initial test on 12/11/2019 was retested on 12/16/2019 using a different methodology and received a negative result. The tests performed each year and their respective results are summarized in Table 1.

**Table 1:** Number of ZKV tests performed by couples undergoing ART procedures, organized by year (2016-2024) and results for IgM and IgG evaluations in females and males.

Year	ZKV tests (IgM female)		ZKV tests (IgG female)		ZKV tests (IgM male)	
	Reagent	Non reagent	Reagent	Non reagent	Reagent	Non reagent
2016	0	0	0	0	0	0
2017	0	1	0	0	0	1
2018	0	0	0	0	0	0
2019	1	9	0	2	0	8
2020	0	32	0	3	0	21
2021	0	181	1	16	0	140
2022	0	262	0	4	0	185
2023	0	81	1	66	0	13
2024	0	2	0	3	0	0
Total	**1**	**568**	**2**	**94**	**0**	**368**
%	**0.18%**	**99.8%**	**2.1%**	**97.9%**	**0.0%**	**100.0%**

Additionally, data from the Brazilian Ministry of Health’s DATASUS system were analyzed and compiled into Graphic 1A and 1B, and are available in Table 2.

**Table 2:** Reported Zika virus cases in São Paulo state and selected municipalities (2016-2024): São Paulo (SP city), São Bernardo do Campo (SBC), São Caetano do Sul (SCS), Santo André (SA), and Diadema (DD).

ZV cases	2016	2017	2018	2019	2020	2021	2022	2023	2024
SP state	10455	1654	1443	3451	1159	964	1104	1783	228
SP state - females	7787	1201	953	2735	864	747	889	1295	185
SP city	843	302	222	329	75	59	54	164	15
SP city - females	588	222	151	228	44	45	28	105	11
SP city - reproductive-age females	386	144	85	152	32	30	16	72	11
SBC	54	3	7	13	6	6	0	2	0
SBC - females	39	1	4	4	4	6	0	0	0
SBC - reproductive-age females	30	1	2	2	3	5	0	0	0
SCS	9	7	5	9	7	6	0	0	0
SCS - females	8	2	1	6	3	2	0	0	0
SCS - reproductive-age females	8	2	1	4	3	1	0	0	0
SA	10	13	3	14	3	0	7	0	1
SA - females	6	9	1	9	2	0	6	0	0
SA - reproductive-age females	4	5	0	5	0	0	4	0	0
DD	0	1	0	0	0	0	0	0	0
DD - females	0	1	0	0	0	0	0	0	0
DD - reproductive-age females	0	1	0	0	0	0	0	0	0

**Graphic 1:**
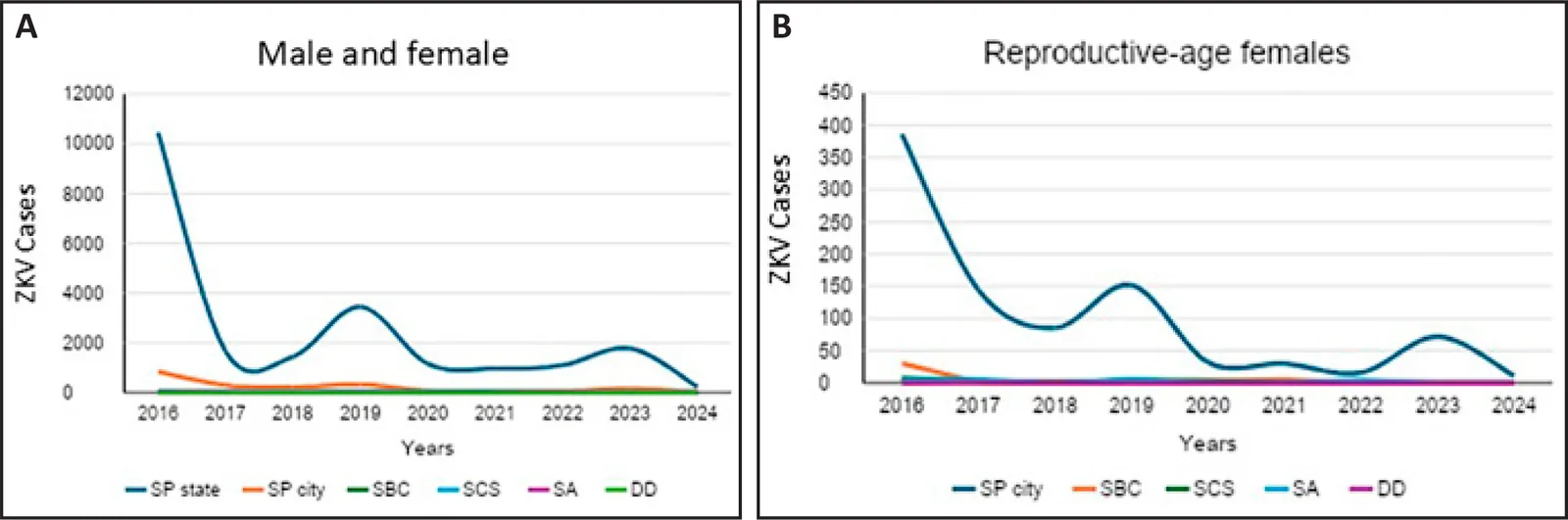
Line chart showing ZKV cases from 2016 to 2024 for SP state (dark blue line) versus SP city (orange line), and ABC cities (São Bernardo do Campo - dark green line, São Caetano do Sul - light blue line, Santo André - purple line, and Diadema - light green line). Data were retrieved from the DATASUS system via the Notifiable Diseases Information System (SINAN 2024), accessed through the TABNET platform from July to August 2024. Part A covers the entire population, while Part B focuses on females of reproductive age (15-39 years old).

## DISCUSSION

The main goal of this study was to determine the presence of ZIKV in a reproductive-age patient group. Data analysis showed a very low rate of ZIKV infection. Specifically, 0.18% of the 569 women undergoing ART tested positive for ZIKV IgM antibodies, while 2.1% of the 96 patients screened for IgG antibodies tested positive. Additionally, no IgM-positive ZIKV cases were found among the 368 male partners in the study. These findings highlight the need to consider mandatory anti-ZIKV testing for people seeking ART or donating biological materials to cell and tissue banks, such as cryopreserved gamete donors, especially given the current pre-screening requirements for oocyte donation and embryo transfer procedures.

The current policy poses significant logistical and financial challenges for healthcare providers, laboratories, and especially patients. These patients are personally responsible for the cost of each test, about US$25 per person, when undergoing ART, increasing their already high treatment expenses. Consequently, patients collectively paid at least US$23,425 for testing alone, as this was not covered by the public health system.

While direct testing costs are significant, the potential clinical repercussions of ZIKV infection are an even greater concern. Projections from the United Nations estimate healthcare expenses for children affected by ZIKV in heavily impacted areas to range between US$500 million and US$5 billion (Nações Unidas Brasil, 2017)

In the general population, ZIKV infection presents with varied clinical manifestations: 50% to 80% of cases are asymptomatic, 20% to 50% have mild illness, and less than 1% develop complications. The incubation period usually ranges from 3 to 14 days, with symptoms such as rash, low-grade fever, joint pain, muscle aches, and conjunctivitis lasting up to 7 days. WHO states that the risk of congenital malformations after maternal ZIKV infection during pregnancy remains uncertain due to differences in study designs and regions, but observational data suggest an estimated 5%-15% risk of Zika-related complications in infants born to infected mothers. These include miscarriage (4% to 7%) and Congenital Zika Syndrome (CZS) (5% to 14%) (Musso *et al*., 2019), with severe microcephaly occurring in 1.5% (CI 0.8%-2.7%) of cases (Pérez *et al*., 2025). CZS covers a range of fetal malformations beyond microcephaly, such as fetal brain disruption syndrome, subcortical calcifications, pyramidal and extrapyramidal signs, congenital contractures, and eye lesions, including chorioretinal atrophy and pigmentary retinopathy. Furthermore, asymptomatic infected neonates may develop mediumand long-term issues, including seizures, difficulty swallowing, hearing loss, and neurodevelopmental delays (de Araújo *et al*., 2018; Krauer *et al*., 2017). Therefore, testing couples of reproductive age during outbreaks in at-risk populations is justified, especially considering the severe consequences of ZIKV infection in pregnant women.

A positive IgM test indicates an active infection at the time of testing, meaning the patient has an ongoing infection when tested. In contrast, a positive IgG test shows that the patient previously encountered the virus and developed immunological memory. In this screening, we identified one IgM-positive patient in December 2019. Retesting this patient five days later resulted in a negative IgM test. For such cases, the protocol recommends repeating the test and waiting through the active infection period before proceeding with reproductive procedures - a protocol followed in this patient’s case. Notably, serological tests for Zika may produce false positives due to possible cross-reactivity with dengue (Brazilian MoH epidemiological bulletin, 2023a). ZIKV tests were conducted from January 2016 through June 2024. The epidemic peak in Brazil occurred from 2015 to 2016. The low IgM positivity rates suggest effective infection control measures and low community transmission during the study period, while the IgG results provide insight into past exposure patterns among women seeking fertility treatment in the region. These findings add valuable data to our understanding of ZIKV epidemiology in São Paulo’s reproductive-age population undergoing assisted reproduction procedures.

A systematic review conducted by Saba Villarroel *et al*. (2024) on the seroprevalence of ZIKV IgG in asymptomatic individuals from January 2000 to July 2023 revealed significant heterogeneity and gaps in the distribution of seroprevalence across different populations worldwide. The rates ranged from 8.4% in Africa to 39.9% in the Americas, with variations among countries within each continent. The estimated pooled global seroprevalence was 21.0%, based on 84 studies involving 63,864 individuals. In this review, the subtropics of Brazil (Santa Maria, Rio Grande do Sul) showed a prevalence of 0.6%, while São Paulo State ranged from 0.1% to 20%.

The declining number of cases over the years and the generally low positivity of ZIKV at the Ideia Fértil facility in São Paulo, Brazil, reflect that surveillance reports show few recent notifications in São Paulo city, including the satellite municipalities of Santo André, São Bernardo do Campo, São Caetano do Sul, and Diadema (according to notification data available through DATASUS). Using SINAN (2024) data to analyze ZIKV occurrence across municipalities has several strengths, such as enabling spatiotemporal monitoring of the disease, identifying vulnerable groups, and supporting public health policy formulation. However, some limitations must be taken into account, including case underreporting, diagnostic challenges due to clinical similarities with other arboviruses (Coelho & Armstrong, 2017), incomplete and inconsistent data entry in reporting forms, delays in data processing, and uneven surveillance quality across municipalities (Brazilian MoH epidemiological bulletin, 2023b).

Considering the low overall positivity rate of ZIKV infection nationwide, testing for ZIKV remains mandatory for all cell and tissue donors, including oocytes and semen, but is optional for patients undergoing ART (RDC 771/2022). The choice to test now depends on each individual patient. Nevertheless, vector control measures, protection against mosquito bites, and avoiding travel to endemic areas or regions experiencing an ongoing epidemic continue to be important recommendations to prevent ZIKV and other arbovirus infections such as Chikungunya and Dengue (Garnica *et al*., 2024).

## CONCLUSION

In this ART patient population from São Paulo State (2016-2024), IgM positivity was rare and IgG positivity was low; however, the results should be interpreted cautiously because the study has a retrospective design, limited IgG sampling, and small positive counts. Patient testing should adhere to current public-health guidance and donor screening regulations (RDC 771/2022). Larger, prospectively designed studies and linkage to municipal-level surveillance are necessary.

## References

[r1] ANVISA - Agência Nacional de Vigilância
Sanitária (Brasil) Resolução da diretoria colegiada - RDC N° 72, de 30 de
março de 2016.

[r2] ANVISA - Agência Nacional de Vigilância
Sanitária (Brasil) Resolução da Diretoria Colegiada - RDC N° 771, de 26 de
dezembro 2022.

[r3] Besnard M, Lastere S, Teissier A, Cao-Lormeau V, Musso D. (2014). Evidence of perinatal transmission of Zika virus, French
Polynesia, December 2013 and February 2014. Euro Surveill.

[r4] Brasil Ministério da Saúde. DATASUS.
OPENDATASUS. Dados de notificações de Zika coletados via Sinan Net e
disponibilizados pelo DATASUS.

[r5] Brazilian MoH epidemiological bulletin (#01) (2023a). Monitoramento dos casos de dengue, febre de chikungunya e febre
pelo vírus Zika até a Semana Epidemiológica 52 de 2022.
Secretaria de Vigilância em Saúde e Ambiente. Braz MoH Epidemiol Bull.

[r6] Brazilian MoH epidemiological bulletin (#13)(2023b) (2023b). Monitoramento das arboviroses urbanas: semanas
epidemiológicas 1 a 35 de 2023. Secretaria de Vigilância em
Saúde e Ambiente. Braz MoH Epidemiol Bull.

[r7] Campos GS, Bandeira AC, Sardi SI. (2015). Zika Virus Outbreak, Bahia, Brazil. Emerg Infect Dis.

[r8] Cardoso CW, Paploski IA, Kikuti M, Rodrigues MS, Silva MM, Campos GS, Sardi SI, Kitron U, Reis MG, Ribeiro GS. (2015). Outbreak of Exanthematous Illness Associated with Zika,
Chikungunya, and Dengue Viruses, Salvador, Brazil. Emerg Infect Dis.

[r9] Coelho FC, Armstrong M. (2017). Tracking the incidence of Zika virus infection using internet
search queries. Epidemiol Infect.

[r10] D’Ortenzio E, Matheron S, Yazdanpanah Y, de Lamballerie X, Hubert B, Piorkowski G, Maquart M, Descamps D, Damond F, Leparc-Goffart I. (2016). Evidence of Sexual Transmission of Zika Virus. N Engl J Med.

[r11] de Araújo TVB, Ximenes RAA, Miranda-Filho DB, Souza WV, Montarroyos UR, de Melo APL (2018). investigators from the Microcephaly Epidemic Research Group;
Brazilian Ministry of Health; Pan American Health Organization; Instituto de
Medicina Integral Professor Fernando Figueira; State Health Department of
Pernambuco. Association between microcephaly, Zika virus infection, and other risk
factors in Brazil: Final report of a case-control study.

[r12] Deckard DT, Chung WM, Brooks JT, Smith JC, Woldai S, Hennessey M, Kwit N, Mead P. (2016). Male-to-Male Sexual Transmission of Zika Virus--Texas, January
2016. MMWR Morb Mortal Wkly Rep.

[r13] Dick GW, Kitchen SF, Haddow AJ. (1952). Zika virus. I. Isolations and serological
specificity. Trans R Soc Trop Med Hyg.

[r14] Foy BD, Kobylinski KC, Chilson Foy JL, Blitvich BJ, Travassos da Rosa A, Haddow AD, Lanciotti RS, Tesh RB (2011). Probable non-vector-borne transmission of Zika virus, Colorado,
USA. Emerg Infect Dis.

[r15] Garnica M, Ramos JF, Machado CM. (2024). Endemic viral infections in immunocompromised hosts: Dengue,
Chikungunya, Zika. Curr Opin Infect Dis.

[r16] Gubler DJ. (2002). The global emergence/resurgence of arboviral diseases as public
health problems. Arch Med Res.

[r17] Gulland A. (2016). Zika virus is a global public health emergency, declares
WHO. BMJ.

[r18] Krauer F, Riesen M, Reveiz L, Oladapo OT, Martínez-Vega R, Porgo TV, Haefliger A, Broutet NJ, Low N, WHO Zika Causality Working Group (2017). Zika Virus Infection as a Cause of Congenital Brain Abnormalities
and Guillain-Barré Syndrome: Systematic Review. PLoS Med.

[r19] MacNamara FN. (1954). Zika virus: a report on three cases of human infection during an
epidemic of jaundice in Nigeria. Trans R Soc Trop Med Hyg.

[r20] Mansuy JM, Dutertre M, Mengelle C, Fourcade C, Marchou B, Delobel P, Izopet J, Martin-Blondel G. (2016). Zika virus: high infectious viral load in semen, a new sexually
transmitted pathogen?. Lancet Infect Dis.

[r21] Musso D, Ko AI, Baud D. (2019). Zika Virus Infection - After the Pandemic. N Engl J Med.

[r22] Musso D, Roche C, Robin E, Nhan T, Teissier A, Cao-Lormeau VM. (2015). Potential sexual transmission of Zika virus. Emerg Infect Dis.

[r23] Nações Unidas Brasil (2017). Custo socioeconômico do zika deve chegar a até US$18 bi na
América Latina e no Caribe.

[r24] Pérez EA, Ticona JA, Alger J, Aranda CA, Niño AA, Ansusinha E, Araújo T, Arias J, Arriaga L, Ávila M, Bardají A, Becerra Mojica C, Benedetti A, Bertozzi A, Bethencourt S, Blackmon K, Borja Aburto V, Brasil P, Brickley E, Britt W (2025). The Zika Virus Individual Participant Data Consortium. Adverse
fetal and perinatal outcomes associated with Zika virus infection during
pregnancy: an individual participant data meta-analysis. EClinicalMedicine.

[r25] Saba Villarroel PM, Hamel R, Gumpangseth N, Yainoy S, Koomhin P, Missé D, Wichit S. (2024). Global seroprevalence of Zika virus in asymptomatic individuals:
A systematic review. PLoS Negl Trop Dis.

[r26] Schuler-Faccini L, Ribeiro EM, Feitosa IM, Horovitz DD, Cavalcanti DP, Pessoa A, Doriqui MJ, Neri JI, Neto JM, Wanderley HY, Cernach M, El-Husny AS, Pone MV, Serao CL, Sanseverino MT, Brazilian Medical Genetics Society-Zika Embryopathy Task
Force (2016). Possible Association Between Zika Virus Infection and
Microcephaly - Brazil, 2015. MMWR Morb Mortal Wkly Rep.

[r27] Venturi G, Zammarchi L, Fortuna C, Remoli ME, Benedetti E, Fiorentini C, Trotta M, Rizzo C, Mantella A, Rezza G, Bartoloni A. (2016). An autochthonous case of Zika due to possible sexual
transmission, Florence, Italy, 2014. Euro Surveill.

[r28] World Health Organization - WHO Zika Virus.

[r29] Zanluca C, Melo VC, Mosimann AL, Santos GI, Santos CN, Luz K. (2015). First report of autochthonous transmission of Zika virus in
Brazil. Mem Inst Oswaldo Cruz.

